# Antioxidant Potential and Oxidative Stress Modulation of *Geranium macrorrhizum* L. Oil Extract in Gentamicin-Induced Nephrotoxicity

**DOI:** 10.3390/ph18091283

**Published:** 2025-08-27

**Authors:** Tsvetelin Georgiev, Galina Nikolova, Viktoriya Dyakova, Silvia Zlateva, Yanka Karamalakova, Ekaterina Georgieva, Kamelia Petkova-Parlapanska, Julian Ananiev, Ana Dobreva, Petya Hadzhibozheva

**Affiliations:** 1 Department of Physiology, Pathophysiology and Pharmacology, Medical Faculty, Trakia University, 6000 Stara Zagora, Bulgaria; 2 Department of Chemistry and Biochemistry, Medical Faculty, Trakia University, 6000 Stara Zagora, Bulgaria; 3 Department of General and Clinical Pathology, Medical Faculty, Trakia University, 6000 Stara Zagora, Bulgaria; 4 Institute for Roses and Aromatic Plants, Agricultural Academy, 6100 Kazanlak, Bulgaria

**Keywords:** *Geranium macrorrhizum* L. oil, gentamicin, murine model, renal protection, antioxidant

## Abstract

**Objectives:** The current study focused on the kidney protection and antioxidant properties along with the potential anti-ferroptotic activity of *Geranium macrorrhizum* L. (*G. macrorrhizum*) oil to ameliorate the acute renal oxidative tissue damage and toxicity of the aminoglycoside antibiotic gentamicin (GM) in an experimental murine model. **Methods:** The research was carried out with mature Balb/c mice distributed into four groups (n = 6). Application of GM (200 mg kg^−1^ intraperitoneal injection for 10 days) was performed to induce kidney injury. Only saline was administered to the controls. The remaining groups were administered *G. macrorrhizum* oil (50 mg kg^−1^ per dose) either used alone or in combination with GM. To assess the renal antioxidant status, the activities of specific antioxidant enzymes, indicators of lipid and DNA peroxidation and renal functional damage were examined using standard commercial kits, ELISA and EPR spectroscopy. **Results:** *G. macrorrhizum* oil analysis revealed 20 organic components belonging to mono- and sesquiterpenoids and long-chain hydrocarbons. The antioxidant and anti-inflammatory effects of *G. macrorrhizum* oil were demonstrated by reduced malondialdehyde, ROS, 8-hydroxy-2′-deoxyguanosine and cytokine levels (especially interleukin-1β) compared with GM. Furthermore, increased activation of superoxide dismutase, catalase and glutathione (GSH) were observed in the kidney homogenates of the animals which received GM in combination with *G. macrorrhizum* oil compared with the GM group. Additional changes in the GSH/glutathione peroxidase-4 axis were detected, suggesting the possible anti-ferroptotic potential of the oil. Nephroprotection was also demonstrated by elevated PGC-1α expression (peroxisome proliferator-activated receptor γ coactivator 1-alpha) and reduced KIM-1 levels (kidney injury molecule-1) following application of the oil. **Conclusions:** The preserved kidney antioxidant and functional properties in the groups treated with oil suggest that *Geranium macrorrhizum* L. could be utilized clinically to mitigate the toxic effects of GM application.

## 1. Introduction

Aminoglycoside antibiotics—gentamicin (GM) in particular—are commonly used to combat different gram-negative infections. They are applied in the treatment of multidrug-resistant tuberculosis and in certain gram-positive infections [1]. In addition to infectious diseases, aminoglycosides are also used for so-called “premature stop codon diseases” such as muscular dystrophy, cystic fibrosis and Rett syndrome [2]. At the same time, GM exhibits significant dose-dependent oto- and nephrotoxicity. Various mechanisms are responsible for GM-induced nephrotoxicity, such as cytotoxic effects on renal tubules, changes in glomerular hemodynamics and inflammation [3]. These alterations are primarily linked to elevated reactive oxygen and nitrogen species (ROS and RNS, respectively) production in tubular and glomerular tissues, which damage proteins, lipids and DNA. ROS and RNS ultimately impair mitochondrial respiration and lead to renal cell death [4]. In this regard, a relatively newly described type of cell death named ferroptosis is of particular interest. Ferroptosis is associated with inactivation of glutathione peroxidase-4 (GPX4), iron accumulation, and subsequent lipid peroxidation, leading to oxidative cell death [5,6]. Currently, there is evidence for the role of the ferroptotic process in the pathogenesis of renal disorders [7,8]. In an acute kidney injury model, ferroptosis inhibition almost completely neutralized tubular cell death and decreased oxidative stress (OS) [9]. Additionally, a study by Zheng et al. [10] showed that ferroptosis plays an important role in neomycin-induced ototoxicity.

In our previous studies [11], we demonstrated that GM-induced renal injury is at least partly attributable to factors that trigger ferroptosis. Natural compounds and antioxidants with proven effects [11,12] could positively influence the functional and morphological renal status of experimental animals. With prolonged use of GM (>7–10 consecutive days), a strategy is needed to reduce adverse effects without compromising antibiotic efficacy [3]. Additive therapy use, alongside GM administration, is an innovative option [13].

*Geranium* species (*Geraniaceae L.*), also known as cranesbills, are flowering perennial herbs encountered throughout Europe and widely used in Balkan traditional medicine [14,15] due to their potent anti-infective and antioxidant activity. Volatile organic compounds, predominantly sesquiterpenes (>70%) and tannins, are considered to contribute to the beneficial result of cranesbill application, expressed as astringent, anti-inflammatory, hypotensive and immunostimulating effects [16].

The most common representatives of the *Geranium* genus are the bigroot cranesbill (*Geranium macrorrhizum* L; *G. macrorrhizum* oil), which are found in moist and shady places. *G. macrorrhizum* oil was found to include many acids (gallic and its derivatives, ellagic, 4-galloyl quinic acids, etc.) as well as quercetin and its glycosides [17]. The antioxidant effects of *G. macrorrhizum* oil can be further attributed to its monoterpene and oxygenated sesquiterpenes components, especially geraniol, β-citronellol, (*E*)-β-elemenone and germacrone, which predominate in the chemical composition. The sesquiterpene germacrone has the highest concentration of essential oils and is a potent ROS and RNS scavenger [18]. The observed antioxidant activities of *G. macrorrhizum* methanol extracts (effectively reducing ferric (II) and cupric (II) ions and scavenging the DPPH and ABTS) were largely attributed to the high phenolic compound concentrations [15].

Bulgarian *G. macrorrhizum* oil is distinguished by its high content of germacrone, capable of exerting ameliorative effects on gastric cancer via modulations of BGC823 cell-cycle-associated protein expression and mitochondria-mediated apoptosis [19]. *G. macrorrhizum* oil has been demonstrated to provide protective effects against acute kidney injury, attributed to the powerful antioxidant and anti-inflammatory activity of quercetin and germacrone [20,21].

In this regard, the widespread distribution of *G. macrorrhizum* L., along with its designation as a non-protected species and its powerful antioxidant properties, makes it a suitable choice for investigation in the area of nephroprotection as well as potential anti-ferroptotic action.

Taking all the above into account, in the current research, our purpose was to evaluate the potential beneficial action of *G. macrorrhizum* oil against GM-induced kidney injury after 10 days of application in a murine model. We hypothesized that the nephroprotective effect of the *G. macrorrhizum* oil application is due to its direct ROS and RNS regulation and anti-inflammatory activity in acute GM nephrotoxicity.

## 2. Results

### 2.1. G. macrorrhizum Oil Chemical Composition

Hydrodistillation of *G. macrorrhizum* L. yielded a colorless to yellowish essential oil. GC FID/MS analysis of the *G. macrorrhizum* oil revealed a total of 20 organic components belonging to the families of monoterpenoids, oxygenated sesquiterpenoids, sesquiterpenoid cycloalkenes, esters and long-chain hydrocarbons. Table 1 summarizes the main components with a relative area greater than 0.1%, along with the retention indices and percentages. Regarding the *G. macrorrhizum* oil composition, the oxygenated sesquiterpenes ((*E*)-β-elemenone (35.3%) and germacrone (20.7%)), along with cycloalkene sesquiterpenes (γ-elemene (4.9%) and germacrene B (3.3%)) were the most abundant compounds in the emission.

### 2.2. Kidney Histopathology

**Figure 1:**
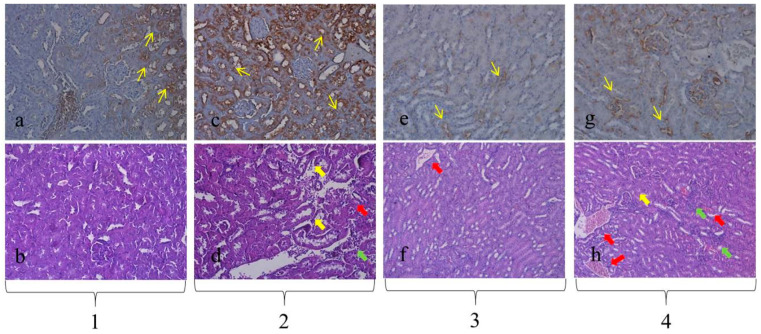
Hematoxylin eosin (HE) and glutathione peroxidase-4 (GPX4) imaging: kidney (200×). 1 = control; 2 = gentamicin (GM)-treated sample; 3 = *G. macrorrhizum* oil-treated sample; 4 = GM + *G. macrorrhizum* oil-treated sample. (**a**) Strong expression of GPX4 (yellow arrow). (**c**) Moderate expression of GPX4 (yellow arrow). (**e**,**g**) Weak expression of GPX4 (yellow arrow). (**b**) Normal appearance, without significant pathological changes. (**d**) Mild degeneration (yellow bold arrow), inflammation (green bold arrow) and vascular congestion (red bold arrow). (**f**) Normal appearance with weak vascular congestion (red bold arrow). (**h**) Weak degenerative (yellow bold arrow) and inflammatory changes (green bold arrow) with vascular congestion (red bold arrow).

At the end of the experimental period, the mice were sacrificed, and their kidneys were collected for histopathological analysis (Figure 1).

We implemented our own protocol to assess pathomorphological changes in distinct groups based on their severity (Table 2). In the kidney tubules of the GM-treated group, mild degenerative and inflammatory changes were observed. No visible changes were detected in the controls. In the group treated only with the extract of *G. macrorrhizum* oil, vascular hyperemia was present. In the combined group, the histopathological changes in the kidney tubules were quite similar to the GM-treated group. Regarding GPX4 expression, the results demonstrated no significant difference between the group treated with GM (moderate expression) and the control one (strong expression). In the *G. macrorrhizum* oil-treated group and GM + *G. macrorrhizum* oil-treated group, the GPX4 expression was also similar (either weak or absent), in sharp contrast to the other two groups.

### 2.3. Analysis of Kidney Injury Molecule-1 (KIM-1), Cystatin C (Cys C) and Glutathione-S-Transferase (GST) in Kidneys

**Figure 2:**
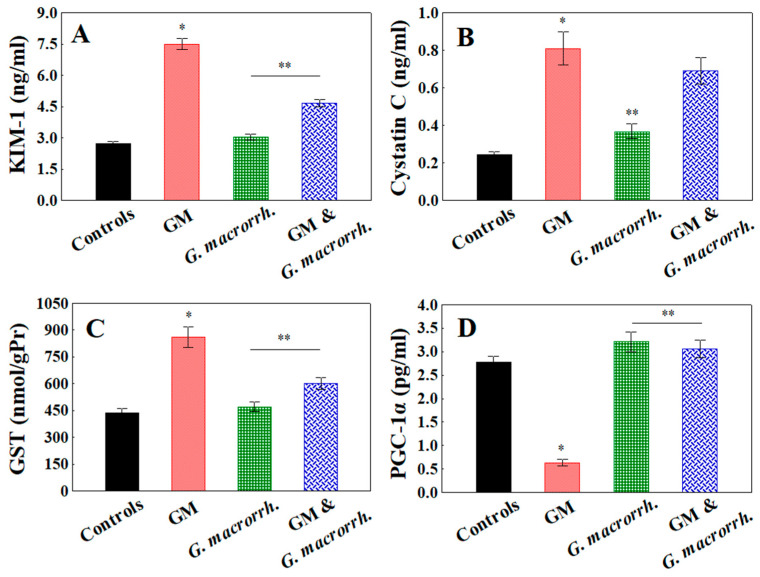
Effects of *G. macrorrhizum* oil application on gentamicin (GM)-induced nephrotoxicity: (**A**) kidney injury molecule-1 (KIM-1); (**B**) cystatin C (Cys C); (**C**) glutathione-S-transferase (GST) ); (**D**) peroxisome proliferator-activated receptor gamma coactivator 1-alpha (PGC-1α). The results are displayed as the mean, including the standard error as a range (n = 6). To identify significant differences, one-way ANOVA with multiple comparisons using Student’s *t*-test were used. * *p* < 0.05 in comparison to the control group. ** *p* < 0.05 compared to the GM administered group.

The renal KIM-1, cystatin C and GST levels were evaluated in order to appraise the functional renal status among the experimental groups and detect the presence of kidney injury (Figure 2). In the GM-treated group, a significant increase in renal KIM-1 expression compared with the control one (7.49 ± 0.26 ng/mL vs. 2.73 ± 0.11 ng/mL, respectively, *p* < 0.05) demonstrated the presence of tubular injury. Similar statistically significant results were obtained when comparing the Cys C expression (0.810 ± 0.09 ng/mL vs. 0.244 ± 0.02 ng/mL, *p* < 0.05) and GST levels (860.90 ± 58.30 nmol/gPr vs. 437 ± 25.30 nmol/gPr, *p* < 0.05) between the GM treated group and the controls, respectively. No significant differences were reported between the group treated only with *G. macrorrhizum* oil and the controls. In the combined GM + *G. macrorrhizum* oil group, KIM-1 expression (4.66 ± 0.18 ng/mL, *p* < 0.05) and GST levels (601.94 ± 31.80 nmol/gPr, *p* < 0.05) were significantly reduced compared with the GM-treated animals. Regarding Cys C expression, a significant difference was not observed (0.690 ± 0.07 ng/mL, *p* > 0.05).

The fibrotic processes triggered by inflammation were examined by assessing the concentration-inactivating collagen (PGC-1α) deposition. Concerning this parameter, the most significant difference between the controls and the GM-treated group was observed. The control values were nearly fivefold higher than those obtained for the GM-treated group (0.63 ± 0.07 pg/mL vs. 2.78 ± 0.12 pg/mL, respectively). The groups with *G. macrorrhizum* oil application demonstrated PGC-1α levels comparable to or exceeding (*p* > 0.05) the controls (3.21 ± 0.21 pg/mL for *G. macrorrhizum* oil and 3.06 ± 0.19 pg/mL for the combination).

### 2.4. Analysis of Hydroxyproline (Hyp), 5-MSL, MDA and 8-OHdG in Kidneys

**Figure 3:**
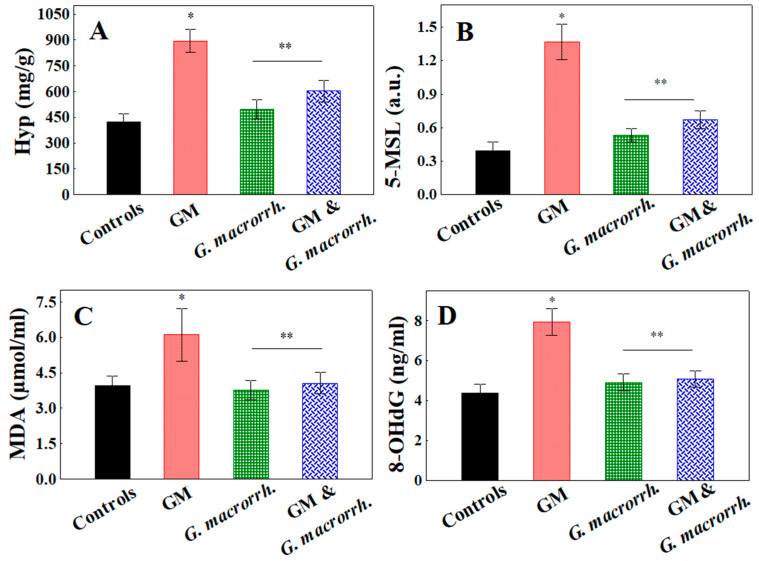
Effects of *G. macrorrhizum* oil application on gentamicin (GM) provoked (**A**) oxidative changes in kidney hydroxyproline content; (**B**) protein oxidation, measured as 3-maleimido proxyl (5-MSL) level; (**C**) lipid peroxidation, evaluated through malondialdehyde (MDA) concentration; and (**D**) DNA oxidation, assessed as 8-hydroxy-2′-deoxyguanosine (8-OHdG) expression. The results are displayed as the mean, including the standard error as a range (n = 6). To identify significant differences, one-way ANOVA with multiple comparisons using Student’s *t*-test were used. * *p* < 0.05 in comparison to the control group. ** *p* < 0.05 compared to the GM administered group.

GM-induced nephrotoxicity was demonstrated by measuring the hydroxyproline (Hyp) content; protein oxidation was measured by 5-MSL-albumin/protein conjugation; DNA oxidation was evaluated by 8-OHdG expression; and lipid peroxidation was measured by the MDA levels in the kidney tissue (Figure 3).

The results demonstrated that GM application statistically significantly increased the Hyp content versus the controls (894.44 ± 65.8 mg/g vs. 421.73 ± 50.2 mg/g, respectively, *p* < 0.05) in the kidney homogenates. Statistical analysis indicated that the Hyp content was significantly lower in the group that was treated with the combination of GM and *G. macrorrhizum* oil (603.21 ± 62.2 mg/g, *p* < 0.05), and the levels were similar to those of the *G. macrorrhizum* oil-treated group (496.90 ± 55.4 mg/g, *p* > 0.05).

Compared with the controls, 5-MSL expression was also significantly increased after GM application (0.39 ± 0.08 vs. 1.37 ± 0.16 a.u., respectively, *p* < 0.05). *G. macrorrhizum* oil significantly reduced GM-provoked protein oxidation (0.67 ± 0.08 a.u., *p* < 0.05). A similar trend was noted concerning the MDA and 8-OHdG concentrations, where GM-treated kidneys exhibited significant elevation in both parameters versus the controls (6.11 ± 1.11 µmol/mL vs. 3.94 ± 0.42 µmol/mL, *p* < 0.05 and 7.96 ± 0.67 ng/mL vs. 4.38 ± 0.46 ng/mL, *p* < 0.05, respectively). Conversely, the *G. macrorrhizum* oil pretreatment significantly reduced the kidney MDA content (4.06 ± 0.45 µmol/mL, *p* < 0.05) and 8-OHdG levels (5.07 ± 0.41 ng/mL, *p* < 0.05) in comparison with GM administration. Simultaneously, the levels were comparable to those of *G. macrorrhizum* oil (3.78 ± 0.41 µmol/mL, *p* > 0.05 for MDA and 4.91 ± 0.42 ng/mL, *p* > 0.05 for 8-OHdG), as well as the controls.

### 2.5. Determination of Renal Oxidative Remodeling

**Figure 4:**
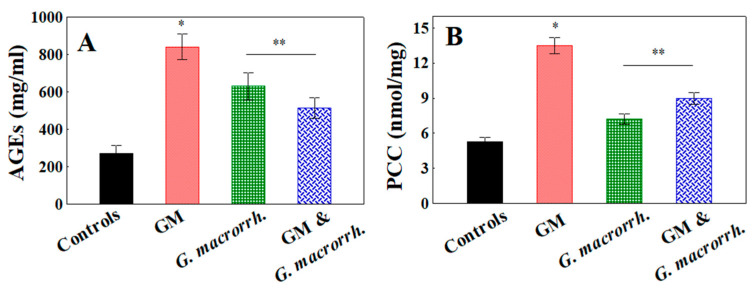
Oxidative remodeling of the kidneys, measured using (**A**) advanced glycation end products (AGEs) in the kidney homogenates and (**B**) the protein carbonyl content (PCC) in the kidney tissue. The results are displayed as the mean, including the standard error as a range (n = 6). To identify significant differences, one-way ANOVA with multiple comparisons using Student’s *t*-test were used. * *p* < 0.05 in comparison with the control group. ** *p* < 0.05 compared with the GM administered group.

Oxidative remodeling in kidneys was determined by measuring the advanced glycation end products (AGEs) and protein carbonylation (PCC) (Figure 4). The group that received GM treatment showed a statistically significant rise in AGE levels relative to the control group (840.4 ± 68.8 mg/mL vs. 269.0 ± 45.2 mg/mL, respectively, *p* < 0.05).

In the groups with the *G. macrorrhizum* oil pretreatment (with GM or alone), the AGEs demonstrated significant reductions compared with the GM group (514.8 ± 55.8 mg/mL for the combined group and 630.1 ± 71.5 mg/mL for the *G. macrorrhizum* extract only group (*p* < 0.05)). In the GM-treated group, the PCC levels were markedly elevated in comparison with the controls (13.48 ± 0.68 nmol/mg vs. 5.29 ± 0.36 nmol/mg, respectively, *p* < 0.05). A statistically significant difference in PCC levels was detected in the animals that received pretreatment with *G. macrorrhizum* oil compared with the GM accumulation model (8.99 ± 0.52 nmol/mg, *p* < 0.05).

### 2.6. Determination of Antioxidant Enzymes

**Figure 5:**
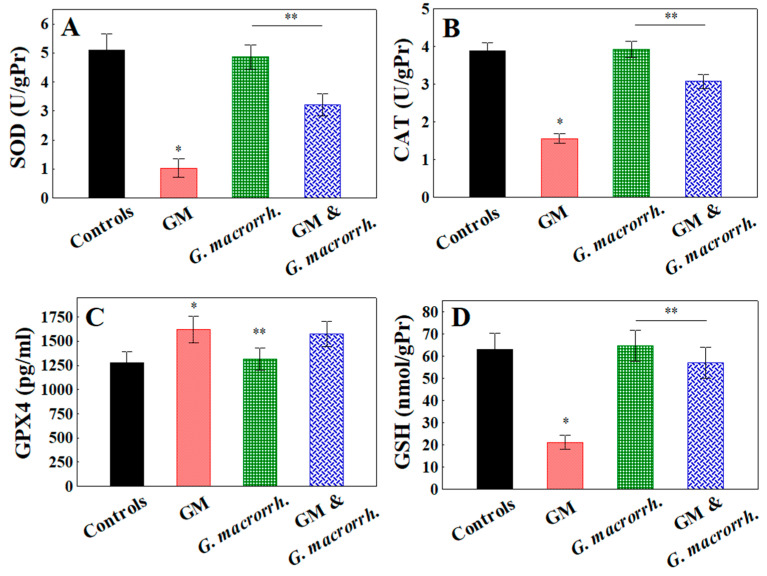
Concentrations of superoxide dismutase (SOD) in kidneys (**A**); catalase (CAT) in kidneys (**B**); glutathione peroxidase-4 (GPX4) in serum (**C**); and glutathione (GSH) in kidneys (**D**). The results are displayed as the mean, including the standard error as a range (n = 6). To identify significant differences, one-way ANOVA with multiple comparisons using Student’s *t*-test were used. * *p* < 0.05 in comparison with the control group. ** *p* < 0.05 compared with the GM administered group.

The antioxidant enzyme expression was affected after GM application (Figure 5). The SOD and CAT activities in the GM-treated group were significantly decreased compared with the controls (1.03 ± 0.32 U/gPr vs. 5.11 ± 0.54 U/gPr, respectively, *p* < 0.05 for SOD and 1.56 ± 0.13 U/gPr vs. 3.89 ± 0.21 U/gPr, respectively, *p* < 0.05 for CAT). No statistically significant difference in SOD and CAT activity was observed between the controls and the *G. macrorrhizum* oil-treated group. Regarding the enzymatic activity in the GM + *G. macrorrhizum* oil pretreatment group, a statistically significant increase was observed compared with the GM-only group (3.21 ± 0.39 U/gPr, *p* < 0.05 for SOD and 3.07 ± 0.19 U/gPr for CAT), with failure of reduction versus the controls and *G. macrorrhizum* oil-only group.

The GPX4 levels measured in mouse serum demonstrated almost comparable levels in the controls and *G. macrorrhizum* oil-only group (1280.7 ± 112.3 pg/mL vs. 1313.3 ± 113.5 pg/mL, respectively, *p* > 0.05). In contrast, GM treatment (with or without protection) demonstrated significant increases in the GPX4 levels versus the controls (1623.3 ± 138.2 pg/mL vs. 1577.7 ± 128.4 pg/mL for the combined group, respectively, *p* < 0.05). Regarding the renal GSH concentration, the results demonstrated the same tendency as that observed for SOD and CAT activity. No statistically significant difference in GSH levels was observed between the controls and the *G. macrorrhizum* oil group. After GM administration, a statistically significant decrease was observed compared with the controls (21.09 ± 3.2 nmol/gPr vs. 63.07 ± 7.40 nmol/gPr, respectively, *p* < 0.05). The GSH levels in the GM + *G. macrorrhizum* oil combination group did not reach the control levels (57.09 ± 7.10 nmol/gPr, *p* > 0.05).

### 2.7. Analysis of Pro-Oxidant Status of the Kidneys

**Figure 6:**
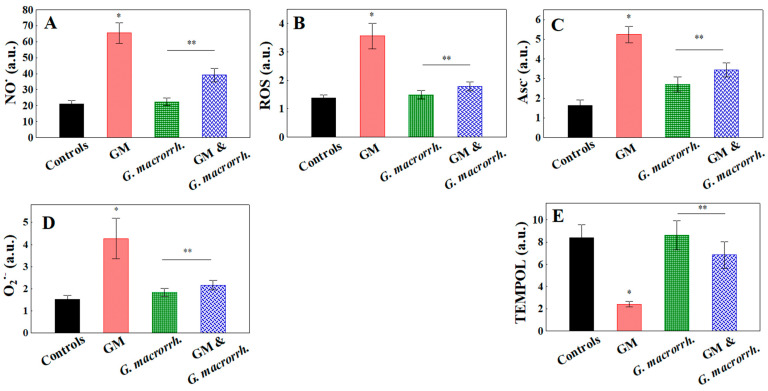
Concentration of free radicals in kidney homogenates: (**A**) nitroxide radicals (NO^•^); (**B**) reactive oxygen species (ROS); (**C**) ascorbate radicals (Asc^•^); (**D**) superoxide anion radicals (O_2_^•−^); and (**E**) 4-Hydroxy-2,2,6,6-tetramethylpiperidine 1-Oxyl (TEMPOL). The results are displayed as the mean, including the standard error as a range (n = 6). To identify significant differences, one-way ANOVA with multiple comparisons using Student’s *t*-test were used. * *p* < 0.05 in comparison to the control group. ** *p* < 0.05 compared to the GM-administered group.

The oxidative stress parameters in the kidneys were investigated by measuring the free radical concentration (ROS production; NO^•^, O_2_^•−^, and Asc^•^), as well as TEMPOL (Figure 6). The GM accumulation model demonstrated a statistically significant increase in all investigated ROS and RNS types (*p* < 0.05) compared with the control. For the NO^•^ radicals, the following results were obtained: 65.50 ± 6.35 a.u. vs. 21.07 ± 2.22 a.u. for the mean values, 3.56 ± 0.45 a.u. vs. 1.37 ± 0.12 a.u. for ROS, 5.24 ± 0.42 a.u. vs. 1.63 ± 0.28 a.u. for Asc^•^ and 4.27 ± 0.92 a.u. vs. 1.53 ± 0.18 a.u. for O_2_^•−^, respectively. Regarding *G. macrorrhizum* oil treatment, the results were comparable with the control group (22.44 ± 2.31 a.u. for NO^•^; 1.49 ± 0.15 a.u. for ROS; and 1.84 ± 0.18 a.u. for O_2_^•−^). The Asc^•^ concentrations were slightly increased (65.50 ± 6.35 a.u.). The GM + *G. macrorrhizum* oil combination demonstrated no statistically significant difference compared with the *G. macrorrhizum* oil group for the ROS (1.78 ± 0.16 a.u.), Asc^•^ (3.44 ± 0.36 a.u.) and O_2_^•−^ (2.16 ± 0.22 a.u.), while the NO^•^ radical levels showed a tendency to rise (39.11 ± 4.11 a.u.).

The TEMPOL spectra in the kidney samples reflect (inverse proportionality) the presence of free radicals. The double-integrated area of the nitroxide spectrum for both the controls and the *G. macrorrhizum* oil treatment group exhibited almost identical values (8.37 ± 1.21 a.u. vs. 8.62 ± 1.32 a.u., respectively, *p* > 0.05).

The TEMPOL intensity in the GM + *G. macrorrhizum* oil combination group was slightly decreased without statistical significance (6.84 ± 1.18 a.u.). As expected, the TEMPOL values in the GM accumulation group showed a sharp, almost fourfold decrease compared with the controls (2.41 ± 0.26 a.u., *p* < 0.05).

### 2.8. Determination of Interleukin Production by Kidneys

**Figure 7:**
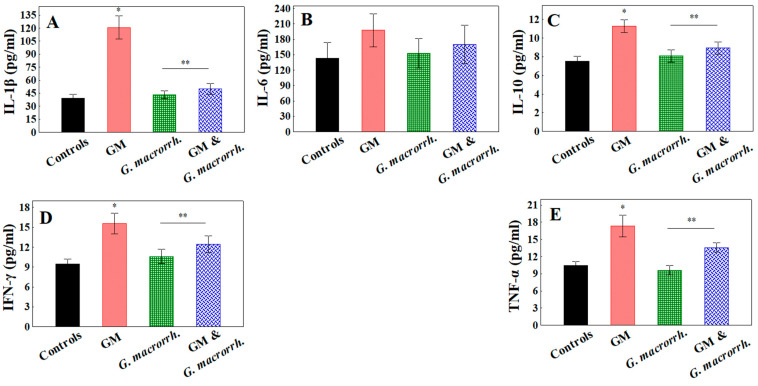
Determination of interleukin (IL) and other cytokines production by kidneys: (**A**) IL-1β; (**B**) IL-6; (**C**) IL-10; (**D**) interferon gamma (IFN-γ); (**E**) tumor necrosis factor-alpha (TNF-α). The results are displayed as the mean, including the standard error as a range (n = 6). To identify significant differences, one-way ANOVA with multiple comparisons using Student’s *t*-test were used. * *p* < 0.05 in comparison to the control group. ** *p* < 0.05 compared to the GM-administered group.

The proinflammatory IL-1β in the renal homogenates demonstrated an almost threefold increase in the GM treated groups compared with the controls (120.7 ± 13.4 pg/mL vs. 39.5 ± 4.2 pg/mL, respectively, *p* < 0.05). The values for both *G. macrorrhizum* oil-treated groups (alone and combined with GM) were similar, being close to the controls (43.4 ± 4.6 pg/mL; 50.1 ± 6.0 pg/mL, respectively) (Figure 7).

Regarding the renal IL-6 levels, although the GM-treated group exhibited the highest values (197.7 ± 32.3 pg/mL), no statistically significant difference was found when compared with the controls (143.6 ± 30.5 pg/mL) or the other groups (152.9 ± 28.6 pg/mL for oil only and and 170.4 ± 37.3 pg/mL for combinations). The IL-10 levels exhibited a similar trend to IL-1β; however, it is noteworthy that the increase observed in the GM accumulation model, in comparison with the controls, was ~1.5-fold (11.27 ± 0.68 pg/mL vs. 7.50 ± 0.56 pg/mL, respectively). The other two groups were similar to the controls and statistically lower than the GM treatment group (8.10 ± 0.66 pg/mL for oil only and 8.93 ± 0.66 pg/mL for combinations, respectively).

GM application demonstrated an almost twofold increase in the INF-γ and TNF-α levels versus the controls (15.57 ± 1.56 pg/mL vs. 9.44 ± 0.77 pg/mL for INF-γ and 17.33 ± 1.92 pg/mL vs. 10.47 ± 0.68 pg/mL for TNF-α, respectively). In relation to the INF-γ concentration in the *G. macrorrhizum* oil-treated groups, the levels were statistically lower than those observed in the GM treatment (10.59 ± 1.08 pg/mL and 12.43 ± 1.28 pg/mL for the combination, respectively). The TNF-α levels in the *G. macrorrhizum* oil group were comparable to the controls (9.61 ± 0.82 pg/mL), and those from the combined group occupied an intermediate position closer to the GM treatment group with statistical difference (13.59 ± 0.81 pg/mL).

## 3. Discussion

GM-induced nephrotoxicity involves different pathways, including receptor-mediated endocytosis in renal tubules, oxidative stress, inflammation, lipid peroxidation, and mitochondrial toxicity [4,22,23]. The histopathological results after 200 mg kg^−1^ of GM administration for 10 experimental days confirmed moderate nephrotoxicity. On the other hand, the disrupted oxidative status balance and mitochondrial dysfunction caused a vicious circle promoting ROS and RNS accumulation, which additionally accelerated kidney damage and death [24,25].

One of the remarkable changes in the GM-treated group in our study was the dramatic increase in ROS and RNS accumulation as a result of exhausted endo- and exogenous antioxidant enzymes. This condition promotes a drastic increase in protein and DNA damage and an elevation in inflammatory cytokines [26]. GM stimulates mitochondrial H_2_O_2_ synthesis and radical production, such as O_2_^•−^, NO^•^ and Asc^•^. As a result, these radicals promote Fe^2+^/Fe^3+^ mitochondrial mobilization [27] and Fenton’s activation with subsequent induction of ferroptosis [28].

Concentrated hydrophobic liquids with volatile aromatic molecules obtained by different methods of extraction, distillation or expression from different parts of the plant have high antioxidant activity [29]. In vivo and in vitro tests of different extracts from *G. macrorrhizum* with ranging concentrations showed that the extracts did not induce significant genotoxicity [30]. For the cranesbill oil, including *G. macrorrhizum* oil, the median lethal dose (LD50) has been determined to be >5000 mg/kg (rat) for oral toxicity, according to Baker and Grant (2018) [31], which makes application of the oil safe for widespread use in cosmetic, food and pharmaceutical industries. In the past, geranium oil has been used to treat inflammation, dysentery and cancer [29]. Natural monoterpenes in geranium oil demonstrate potent chemopreventive effects in skin cancer via stepwise reduction of inflammation, oxidative stress and tumorigenesis and the inhibition of key apoptotic pathways [32]. Geranium oil has been shown to modulate neurodegenerative diseases through its anti-inflammatory properties [33]. The detoxifying ability of geranium oil provides the body with antioxidant protection [34] and effective fungicidal activity against *Mucor mucedo—Aspergillus*-resistant species [35].

Different reports [36,37] commented that geranium oil components often act synergistically, combining and enhancing the overall antioxidant response against ROS and RNS production [38,39] and oxidative stress changes.

Considering these facts, our objective was to determine for the first time the potential properties of *G. macrorrhizum* oil (50 mg kg^−1^, p.o.) extract to reduce renal inflammation and lesions and modulate oxidative disorders in rodents exposed to acute or progressive GM-induced nephrotoxicity.

GC FID/MS analysis revealed the maximum exposure of oxygenated sesquiterpenoids, namely (*E*)-β-elemenone (35.3%) and germacrone (20.7%), together with cycloalkene sesquiterpenes, namely γ-elemene (4.9%), the main components in the used *G. macrorrhizum* oil. Our findings align with those of Chalchat et al. (2002) [40] and Ameline et al. (2023) [41], who examined the quantity and chemical composition of *G. macrorrhizum* oils, identifying β-elemenone and germacrone as the primary components. In addition, Ilić et al. (2020) [16] emphasized the uniqueness of the chemical constitution of volatile organic compounds in the *G. macrorrhizum* composition, due to the high sesquiterpene content (>70%). Germacrone and β-elemenone are terpenes with ketone function, and this group of compounds exhibits strong ketone inhibitory activity on acetylcholinesterase [42].

Furthermore, germacrene has been identified in three isomers—germacrene A, germacrene B and germacrene D (0.3–3.3% respectively)—in our sample. Bulgarian *G. macrorrhizum* oil shows a stronger DPPH scavenging potential and, according to Pearson’s correlation, the strongest Germacrene A/D effect, which is about 92.3% [18].

The observed ROS and RNS reduction in the groups having received *G. macrorrhizum* oil could be explained by the well-documented protective effects against acute kidney injury of quercetin and germacrone [20,21], stemming from their antioxidant and anti-inflammatory properties. The antioxidant properties of *G. macrorrhizum* oil can primarily be attributed to its monoterpene and sesquiterpene constituents, which are prevalent in its chemical composition [37].

Kashyap et al. (2016) [43] documented direct ROS and RNS scavenging, metal ions chelation and lipid peroxidation inhibition in addition to an impact on Nrf-2 expression. These findings suggest that *G. macrorrhizum* oil works as a ferroptotic inhibitor. Evidence of this was reported by our team’s own findings of the complete recovery of MDA and 8-OHdG levels, which are biomarkers indicative of oxidative nuclear and mitochondrial DNA damage [44], in the groups protected by *G. macrorrhizum* oil. Similar results were obtained by Wang et al. (2023) when investigating germacrone’s effects on renal tubular cells and apoptotic inhibition [45]. Although we hypothesize that the primary pathway by which *G. macrorrhizum* oil inhibits ferroptosis is SLC7A11 or a more complex path, the specific molecular mechanisms remain unclear.

The evidence that *G. macrorrhizum* oil ameliorates SOD and CAT enzyme activities in kidneys further supports its role in restoring oxidative homeostasis. Geranium oil’s antioxidant properties have an ability to recover GSH depletion. According to Ragab (2007) [46], geranium extract exhibits a cytoprotective effect against gamma radiation. Decreasing GSH is a basic step for cellular protection from oxidative stress [47]. Boadi et al. (2016) [48] also documented oxidative status restoration, particularly the restoration of GSH and SOD activity, following quercetin application. In a diabetic nephropathy model, Zhuang et al. (2021) [49] reported that germacrone upregulates Nrf-2 expression and promotes GSH, SOD and glutathione peroxidase activity.

Similar effects and activation of the GPX4 axis by germacrone were reported by Jin et al. (2022) [50]. We suppose that the serum GPX4 levels in the GM treated group were increased because of enzyme release from damaged renal cells. The same trend observed in the GM + *G. macrorrhizum* oil group was probably the result of a complex response, namely systemic compensation due to activation of the body’s antioxidant system following GM-incurred damage and concurrent *G. macrorrhizum* oil application. Additionally, the low GPX4 kidney expression in the *G. macrorrhizum* oil groups may be associated with other properties of the oil that affect renal GPX4. Zhao et al. (2024) [51] reported that β-elemene, a sesquiterpene extracted from the essential oil of Curcuma plants, promotes ferroptosis and inhibits GPX4 expression. Sesquiterpenoids that are structurally related to β-elemene, such as γ-elemene and β-elemenone, were abundantly identified in the essential oil of *G. macrorrhizum* that we used. These findings could explain the low GPX4 expression in histological samples, which occurred concurrently with an enhanced oxidant status in the groups treated with *G. macrorrhizum* oil. These results are also in accordance with those of Xie et al. (2024) [52] and Li et al. (2022) [53] concerning gallic and ellagic acid, which are compounds in some geranium plant extracts. Serum GPX4 expression along with the absence of histological improvement between the GM group and the combination group indicated that the oil content requires further purification in order to improve its protective properties and elimination of compounds with controversial effects.

Another sign of an acute kidney injury is greater Cys C presence and KIM-1 levels after GM application. Cys C expression in blood and renal cells serves as a significant indicator of the glomerular filtration rate, reflecting kidney function [54]. The expression of KIM-1 is commonly elevated in cases of both acute and chronic renal failure, with its levels being directly linked to the extent of kidney injury and the presence of fibrosis [55,56]. The incomplete Cys C recovery in the GM + *G. macrorrhizum* oil group confirms the histological findings and indicates that certain degenerative and inflammatory processes still persist. However, the KIM-1 reduction in the GM + *G. macrorrhizum* oil group demonstrates improvement of the proximal tubular part of the nephron. A reduction in hydroxyproline levels, a biomarker indicating kidney fibrosis, serves as confirmation of an ameliorating effect [57]. The same applies to 5-MSL, which is responsible for protein modification, particularly in relation to albumin injuries, through its binding to sulfhydryl (-SH) groups [58] after *G. macrorrhizum* oil pretreatment.

Additional OS indicators, possibly correlating with kidney function and recovery, include biomarkers related to the oxidative products of proteins and lipids (AEGs and PCC). Through lipid oxidation reduction and ROS and RNS scavenging, the active *G. macrorrhizum* oil compounds drive the recovery mechanism of the renal tubular system. There is evidence that germacrone modulates ferroptosis in the kidney’s tubular cells by stimulating mitophagy and blocking mtDNA-STING signaling [45]. The mtDNA-STING signaling pathway triggers an immune response. This finding corresponds to our results, where we observed a decrease in the level of proinflammatory substances triggered by the OS and mitochondrial damage.

The most pronounced decrease was observed in the levels of IL-1β, but IL-6, IFN-γ and TNF-α were also recovered significantly. There is evidence that germacrone not only reduces the levels of pro-oxidants but also lowers the levels of some pro-inflammatory interleukins [49], like IL-1β, IL-6 and INF-γ. TNF-α and IL-6 reduction in the *G. macrorrhizum* oil groups is associated not only with an anti-inflammatory effect but also an anti-fibrotic one. Ibrahim et al. (2022) [59] indicate that the referenced interleukins, through mast cell activation, have the potential to enhance fibroblast activity. This finding is consistent with the reports of Li et al. (2022) [53] regarding gallic and ellagic acid. In our study, GM application for 10 days resulted in elevated IL-10 levels while significantly suppressing PGC-1α.

PGC-1α is closely associated with mitochondrial function and plays a crucial role in sustaining energy metabolism [60]. A deficiency in PGC-1α triggers an inflammatory response in the kidneys, which is exacerbated during episodes of acute renal injury [61]. These findings, in conjunction with the results of our study, illustrate that GM-induced suppression of PGC-1α levels intensifies renal stress, advances nephrotoxicity progression and impacts cell survival. Additionally, GM has also triggered a compensatory reaction due to activation of the STAT5 signaling pathway and induction of IL-10 expression [62,63]. These findings lead to the hypothesis that GM probably stimulates both M1 and M2 inflammatory and healing responses, but the pro-inflammatory response appears to be more dominant. *G. macrorrhizum* oil protection inhibits both responses, which explains the full recovery of PGC-1α expression in kidney tissue and, in parallel, the decreased IL-10 levels.

For a nitroxide spin probe, TEMPOL exhibits significant SOD mimetic activity and is often used for direct assessment of ROS and the total OS. Similar to SOD, it catalyzes the dismutation process of O_2_^•−^ to H_2_O_2_, and oxygen and can accumulate in the cell, effectively reducing superoxide anion radicals [58]. In this manner, it could ameliorate oxidative stress-mediated renal dysfunction and glomerular injury [11,64]. The independent application of *G. macrorrhizum* oil had no pro-oxidant effect, which does not alter the redox status in kidney tissue. In contrast, GM-induced kidney injury led to overproduction of free radicals and severe oxidative stress. This could explain the observed pattern in our experiment concerning the lowest TEMPOL values in the GM-treated group, while in the protected group, the levels of TEMPOL were similar to the control ones. This tendency corresponded to the levels of free radicals, particularly ROS and O_2_^•−^, measured in the kidney homogenates. Our results provide direct biochemical evidence for the nephroprotective effect of *G. macrorrhizum* oil against gentamicin-induced OS in the kidneys within this toxicological model.

## 4. Materials and Methods

### 4.1. Plant Material

The aerial and underground parts (leaves, flowers and roots) of *G. macrorrhizum* (Geraniaceae) were collected in October 2022 from the experimental field at the Institute for Roses and Aromatic Plants in Kazanlak, Bulgaria. *G. macrorrhizum* essential oil was obtained via hydrodistillation with laboratory equipment with a 5-L vessel. The resulting distillate was subjected to redistillation, and the oils obtained from the two stages were mixed at their natural ratio.

### 4.2. Gas Chromatography-Mass Spectrometric Analysis (GC FID/MS)

The chemical composition of the *G. macrorrhizum* oil was determined through gas chromatography with a flame ionization detector (FID). A GC system (Agilent 7820A; Agilent, Santa Clara, CA, USA) coupled with a flame ionization detector (5977B MS) was used. The EconoCapTM ECTM-5 capillary column (30 m × 0.25 mm (ID) × 0.25 μm film thickness) was employed for analytics separation. The column temperature was programmed to range from 65 °C to 230 °C at a heating rate of 1 °C/min for the GC System. The detector and injector temperatures were set at 250 °C. The injected sample volume was 1.0 µL in flow split mode (100:1). The compounds were identified by comparing the retention times and relative Kovacs indices (RIs) with those of standard substances and mass spectral data from the NIST’08 (National Institute of Standards and Technology, USA) and Adams libraries.

### 4.3. Animals, Experimental Design and Ethical Approval

Twenty-four male Balb/c mice aged 7 weeks (average weight: 31.5 ± 4.5 g) were obtained from the Institute of Animal Science in Slivnitsa, Bulgaria. The animals were kept in an environment with a regulated temperature (21 °C) and humidity (52%) and a 12-h dark/light cycle over a 10-day period for adaptive feeding and acclimatization (light phase: 7:00 a.m.–7:00 p.m.) in accordance with the license number (317/6000-0333/9 December 2021), which complies with Directive 2010/63/EU regarding the protection of animals used for experimental and other scientific purposes. Throughout the experiment, the mice were provided with ad libitum access to fresh water and received a basal diet that contained 19.6% protein, 4.03% fat, 6.89% fiber, 10.71% moisture and 8.97% ash.

The GM-induced nephrotoxicity murine model was developed with daily IP injections of 200 mg kg^−1^ day^−1^ for 10 consecutive days, according to previous models [23,65]. GM was purchased in pharmacy form.

The plants from the Geranium family have shown medicinal properties at different doses. These pharmacological effects vary depending on the concentrations of the chemical components [34,66,67]. Based on different results from in vitro and in vivo studies [15,34,66,67,68] concerning the antioxidant activity and cytoprotective effect of Geranium family extracts, 50 mg kg^−1^ *G. macrorrhizum* oil was used in the current research.

Four groups of experimental animals were formed (n = 6), which were as follows:

(1) The control group was administered via oral gavage 0.1 mL of isotonic NaCl solution (0.9%) for 10 days;
(2) The gentamicin (GM)-induced nephrotoxicity group’s animals received GM (administration of GM 200 mg kg^−1^ day^−1^ i.p.) for a duration of 10 days;
(3) The *G.macrorrhizum* oil-only group’s animals were treated with a dose of 50 mg kg^−1^ day^−1^ b.w., p.o. for 10 days;
(4) The GM + *G.macrorrhizum* oil combination group’s animals were treated with *G. macrorrhizum* oil (50 mg kg^−1^ day^−1^, p.o.) and GM (200 mg kg^−1^ day^−1^, i.p.) for 10 days.

The *G. macrorrhizum* oil was mixed with d.H_2_O and refined olive oil (Lekkas Farm, Mikro Horio, Greece). Daily monitoring of the physiological condition and behavior of the experimental animals was conducted.

The animals were euthanized under anesthesia (Nembutal, 50 mg kg^−1^, i.p.) on the 11th day of the experiment. Blood samples were obtained using the standard intracardiac technique, and fresh blood was gathered in vacutainer serum tubes. Serum samples were prepared through centrifugation (4000 rpm, 10 min at 4 °C). The kidneys of the mice were measured, with the right kidney being maintained in ice-cold 0.05 M PBS (pH = 7.5; 4 °C), homogenized independently and subsequently analyzed. The left kidney was kept in 10% formalin buffer for histological analysis.

### 4.4. Histopathological Analysis

The kidney tissue was embedded in paraffin following perfusion, underwent dehydration through a graded series of ethanol and was fixed in 10% phosphate-buffered formalin for a duration of 24 h. The kidney tissues (sliced into 5-μm sections) were placed on gelatin-coated slides, dewaxed twice using xylene and rehydrated through a series of decreasing ethanol concentrations. The histological assessment was conducted after staining the sections with a standard hematoxylin/eosin-based method (0.1% H&E) to identify notable kidney injuries.

Immunohistochemistry was performed as follows. The tissue specimens were fixed in 10% buffered formalin and embedded in paraffin. The next steps were dewaxing and blocking endogenous peroxidase for 5 min with a blocking reagent according to the protocol. Then, the slides were washed three times with PBS and incubated with a primary antibody for 1 h. After that, the slides were washed three times, incubated with marked polymer and then washed again. In the last phase, they were incubated with DAB substrate-chromogen and washed again. At the end, they were contrastained with Mayer’s hematoxylin. The antibodies used were monoclonal mouse anti-human GPx-4 (E-12) (sc-166570, Santa Cruz Biotechnology, Dallas, TX, USA) at a primary antibody 1:50 dilution. The immunostaining kit detection system used was an EnVision™ FLEX+, Mouse, High pH, (Link) (K8002, DAKO, Nowy Sącz, Poland).

### 4.5. Renal Hydroxyproline (Hyp) Measurement

Renal hydroxyproline (Hyp) measurement, used to quantify kidney damage and measure the tissue collagen content indirectly, was conducted spectrophotometrically at 550 nm of absorption with the Woessner method as previously described [12] and presented in terms of milligrams of Hyp per gram of tissue.

### 4.6. Electron Paramagnetic Resonance (EPR) Measurement of Oxidative Stress

All EPR analyses were conducted using fivefold measurement in the recorded spectra with the following characteristics: 3503–3515 G center field; 6.42–20.00 mW microwave power; 5–10 G modulation per sample and 1–5 scans per sample.

#### 4.6.1. Renal ROS Production

To examine ROS production in the kidneys, EPR spectroscopy was used in conjunction with *N*-tert-butyl-alpha-phenylnitrone (PBN) serving as a spin-trapping agent, in accordance with our modified methodology [69]. In brief, 100 μL of homogenized kidney tissue was combined with 900 μL (50 mM) PBN dissolved in dimethyl sulfoxide (DMSO). The mixture underwent centrifugation at 4000× *g* for 10 min at a temperature of 4 °C, with the results presented in a.u.

#### 4.6.2. Renal Nitric Oxide (NO^•^), Superoxide (O_2_^•−^) and Ascorbate (Asc^•^) Radical Generation

We have applied a modified EPR method to estimate the NO^•^ radical levels. The method is based on NO^•^ production proportional to the spin–adduct formed between the spin trap carboxy 2-(4-carboxyphenyl)-4,4,5,5-tetramethyl (CPTIO.K) and NO^•^ in renal samples [11]. CPTIO.K (50 μM) was dissolved in 50 mM Tris (pH = 7.5) and DMSO (9:1), centrifuged (4000× *g* for 10 min, 4 °C). Afterwards, renal samples (100 μL) were added in CPTIO.K in 1:1 ratio, and spin–adducts were measured; results are presented in arbitrary units (a.u.).

The superoxide (O_2_^•−^) levels in renal samples were assessed in relation to the spin-adduct generated using the spin-trap CMH (1-hydroxy-3-methoxycarbonyl-2,2,5,5-tetramethylpyrrolidine), following established methods [70,71]. Kidney tissue (30 μL) was activated in CMH (with ratio 1:1), placed in an ice bath, and incubated for 5 min; results are displayed in arbitrary units (a.u.).

The ascorbate (Asc^•^) radicals were studied according to the methods of Bailey (2004) [72]. Briefly, the kidney tissue was prepared in DMSO (1:3 ratio) and centrifuged (4000 rpm for 10 min 4 °C). The supernatants were directly analyzed and presented in arbitrary units (a.u.).

#### 4.6.3. Renal 3-Maleimido Proxyl (5-MSL) Protein Oxidation

The extent of protein and albumin impairment in the renal samples was evaluated through the in vivo EPR technique utilizing spin conjugation with 3-maleimido proxyl (5-MSL). Kidney samples weighing 10 mg were combined with 0.9 mL, 20 mM 5-MSL, dissolved in DMSO and centrifuged (1000 rpm for 15 min, 4 °C). The content of protein and albumin (free SH group malformations) was expressed in arbitrary units (a.u.), following a previously outlined method [12].

#### 4.6.4. 4-Hydroxy-2,2,6,6-Tetramethylpiperidine 1-Oxyl (TEMPOL)

A TEMPOL radical (50 µL at 2 mM) was introduced into the kidney homogenates, mixed for 5 s at 23 °C and incubated for 10 min. Each sample underwent two scans which were repeated, following the methodology of Georgieva et al. (2023) [58], with the results expressed in arbitrary units (a.u.).

### 4.7. Renal Protein Carbonyl Content (PCC) and Advanced Glycation End Products (AGEs)

Oxidative impairment of proteins was evaluated by measuring the reaction of dinitrophenylhydrazine (DNPH) with carbonyl groups to form DNP hydrazone (2 h at 37 °C), as determined using an OxiSelect Total Carbonyl Protein ELISA Kit (Cell Biolabs, San Diego, CA, USA). PCC was established using oxidized and reduced BSA standards at an absorption wavelength of 370 nm, with carbonyl derivatives quantified in terms of nanomoles per milligram.

The AGE levels were monitored in a similar manner to PCC using an OxiSelect AGE competitive ELISA Kit (Cell Biolabs, San Diego, CA, USA). The assessment of the AGE-protein content in the unidentified samples was carried out via comparison to a previously established standard AGE-BSA curve and measurement in nmol/mg.

### 4.8. Kidney Functional Parameters

Kidney functional damages were assessed by commercial kits used to detect cystatin C (CysC), kidney injury molecule-1 (KIM-1; No.MBS175125), glutathione-S-transferase (GST), and concentrations of gamma-glutamyl-transpeptidase (gamma-GT) in serum. PGC-1α levels were evaluated using ELISA kits (G-Biosciences, St. Louis, MO, USA).

### 4.9. Renal Lipid Peroxidation and Endogenous Antioxidant Activity

Lipid peroxidation in renal samples was evaluated using a previously described method [73], comparing it to equivalent concentrations of malondialdehyde (MDA nmol/mg protein; THERMO Sci., RS232C, Waltham, MA, USA). Renal catalase (CAT), superoxide dismutase (SOD) activity and GSH levels were assessed using previously outlined techniques [12].

### 4.10. Measurement of Pro-Inflammatory Parameters in Renal Tissue and Serum

The levels of cytokines (IFN-γ, TNF-α, IL-1β, IL-10 and IL-6) were evaluated using ELISA kits (G-Biosciences, St. Louis, MO, USA).

### 4.11. Statistical Analysis

EPR measurements were conducted at ambient temperature using a Bruker BioSpin GmbH (Ettlingen, Germany), which was equipped with a standard resonator. The EPR analysis was conducted utilizing WIN-EPR SimFonia 1.2/6130860 software, version V2.00Rev.03 (2017). Spectral processing was conducted using Bruker WIN-EPR SimFonia 1.2/6130860 software after double integration, and the results are presented in arbitrary units (a.u.).

The analysis of the results was performed with Statistica 8.0 (StatSoft, Inc., Tulsa, OK, USA) and the results are displayed as the mean, including the standard error as a range. To identify significant differences, one-way ANOVA with multiple comparisons using Student’s *t*-test were used. A *p* value of less than 0.05 was considered to be statistically significant.

### 4.12. Limitations of the Study

The results of this study demonstrate that *G. macrorrhizum* oil ameliorates kidney dysfunction caused by GM treatment. Nonetheless, it is important to acknowledge certain limitations that may have affected the overall impact of oil on GM-provoked kidney injury. Considering ethical factors, the number of animals studied in each group was restricted to six. This amount was sufficient to reveal the most significant differences; however, it may not have been enough to identify further correlations among certain parameters. The results indicate significant changes in most investigated functional kidney biomarkers, as well as correlation between them and the inflammatory and degenerative parameters. However, a more detailed exploration of these markers using pathway-specific inhibitors or genetic methodologies would enhance the understanding of their role in the process. Additionally, inclusion of more precise analysis like qPCR for the measurement of pro-inflammatory markers and the use of ImageJ to provide blinded quantitative assessments could improve the strength of the results. Further investigations to reveal whether the nephroprotective effects of *G. macrorrhizum* oil are ferroptosis-specific and determine the molecular mechanisms by which the plant oil affects ferroptosis will be necessary. In the current research, the in vivo GM activity was not examined through the co-administration of *G. macrorrhizum* oil, which would also provide further insights into the antimicrobial characteristics of the combination therapy. Additional research is required to determine potential dosages that may positively influence kidney toxicity with prolonged use, particularly in ferroptosis protection mechanisms, as well as examining the molecular aspects of DNA damage.

## 5. Conclusions

In the current research, we documented a notable increase in oxidative stress due to increased production of ROS and pro-inflammatory cytokines, as well as decreased redox potential, including in the GSH/GPX axis, accompanied by impaired kidney function and structural damage in the GM-accumulated rodent model. *G. macrorrhizum* oil (50 mg kg^−1^, p.o.) administration restored most of the parameters related to oxidative stress, inflammation and kidney damage. The presented results suggest that the reduction in GM-induced kidney injury enhances the oxidative balance, including the GSH/GPX4 axis, and also has an influence on kidney cell survival. Additionally, a well-established anti-inflammatory action of the oil was observed, reflecting a significant impact on the levels of pro-inflammatory cytokines. *Geranium macrorrhizum* oil serves as a potent natural complex antioxidant and anti-inflammatory agent that may be used as a type of complementary therapy against gentamicin-induced nephrotoxicity.

## Figures and Tables

**Table 1:** Characteristics of the *G. macrorrhizum* oil chemical compounds.

№	Compound or Family	MS Sim.	RI Ref.	RI Exp.	Rel. %
	Monoterpenes				
1	α-Pinene	93	933	935	tr
2	β-Pinene	96	978	975	0.1
3	Mycrene	96	991	989	0.1
4	*p*-Cymene	97	1020	1018	0.5
5	Limonene	98	1024	1022	0.2
6	(*E*)-β-Ocimene	95	1046	1041	0.2
7	γ-Terpinene	97	1058	1058	0.5
	Sesquiterpenes/cycloalkene class				
8	Terpinolene	97	1086	1083	0.4
9	δ-Elemene	92	1335	1340	tr.
10	β-Elemene	93	1390	1390	1.4
11	Italicene	97	1410	1403	0.1
12	γ-Elemene	91	1432	1431	4.9
13	γ-Curcumene	95	1482	1482	0.9
14	Germacrene D	94	1485	1484	0.5
15	Germacrene A	97	1511	1514	0.2
16	Germacrene B	97	1557	1552	3.3
	Oxygenated sesquiterpenes				
17	(*E*)-β-Elemenone	92	1600	1601	35.3
18	Germacrone	90	1698	1695	20.7
	Long-chain alkane hydrocarbon				
19	*n*-Nonadecane	97	1900	1901	0.3
20	*n*-Heneicosane	97	2100	2100	0.1

Legend: Relative abundance was expressed as the mean value ± SD of seven replicates. RI exp. = experimental retention index; RI ref. = referenced retention index; Rel. % = relative percent; MS Sim. = mass spectral similarity.

**Table 2:** Comparative pathomorphological changes among the groups: Control, GM-treated group, *G. macrorrhizum* oil-treated group, GM + *G. macrorrhizum* oil combination.

Groups (n = 6)	GPX4	Degeneration	Necrosis	Inflammation	Hyperemia
Control	3+	0	0	0	0
GM (200 mg kg^−1^)	2+	1	0	1	1
*G. macrorrhizum* oil (50 mg kg^−1^)	0/1+	0	0	0	1
GM (200 mg kg^−1^) + *G. macrorrhizum* oil (50 mg kg^−1^)	0+	0/1	0	0/1	1

Legend: 0 = no changes; 1 = weak changes; 0+ = no expression; 1+ = weak expression; 2+ = moderate expression; 3+ = strong expression.

## Data Availability

The contributions originally presented in this study are incorporated within the article. Any further inquiries may be directed toward the corresponding authors.

## References

[1] Garneau-Tsodikova S., Labby K.J. (2016). Mechanisms of Resistance to Aminoglycoside Antibiotics: Overview and Perspectives. MedChemComm.

[2] Greber B.J., Ban N. (2016). Structure and Function of the Mitochondrial Ribosome. Annu. Rev. Biochem..

[3] Randjelovic P., Veljkovic S., Stojiljkovic N., Sokolovic D., Ilic I. (2017). Gentamicin nephrotoxicity in animals: Current knowledge and future perspectives. EXCLI J..

[4] Gamaan M.A., Zaky H.S., Ahmed H.I. (2023). Gentamicin-induced nephrotoxicity: A mechanistic approach. Azhar Int. J. Pharm. Med. Sci..

[5] Dixon S.J., Lemberg K.M., Lamprecht M.R., Skouta R., Zaitsev E.M., Gleason C.E., Patel D.N., Bauer A.J., Cantley A.M., Yang W.S. (2012). Ferroptosis: An iron-dependent form of nonapoptotic cell death. Cell.

[6] Li C., Deng X., Xie X., Liu Y., Friedmann Angeli J.P., Lai L. (2018). Activation of Glutathione Peroxidase 4 as a Novel Anti-inflammatory Strategy. Front. Pharmacol..

[7] Friedmann Angeli J.P., Schneider M., Proneth B., Tyurina Y.Y., Tyurin V.A., Hammond V.J., Herbach N., Aichler M., Walch A., Eggenhofer E. (2014). Inactivation of the ferroptosis regulator Gpx4 triggers acute renal failure in mice. Nat. Cell Biol..

[8] Linkermann A., Skouta R., Himmerkus N., Mulay S.R., Dewitz C., De Zen F., Prokai A., Zuchtriegel G., Krombach F., Welz P.S. (2014). Synchronized renal tubular cell death involves ferroptosis. Proc. Natl. Acad. Sci. USA.

[9] Martin-Sanchez D., Ruiz-Andres O., Poveda J., Carrasco S., Cannata-Ortiz P., Sanchez-Niño M.D., Ruiz Ortega M., Egido J., Linkermann A., Ortiz A. (2017). Ferroptosis, but Not Necroptosis, Is Important in Nephrotoxic Folic Acid-Induced AKI. J. Am. Soc. Nephrol..

[10] Zheng Z., Tang D., Zhao L., Li W., Han J., Hu B., Nie G., He Y. (2020). Liproxstatin-1 Protects Hair Cell-Like HEI-OC1 Cells and Cochlear Hair Cells against Neomycin Ototoxicity. Oxid. Med. Cell. Longev..

[11] Georgiev T., Nikolova G., Dyakova V., Karamalakova Y., Georgieva E., Ananiev J., Ivanov V., Hadzhibozheva P. (2023). Vitamin E and Silymarin Reduce Oxidative Tissue Damage during Gentamycin-Induced Nephrotoxicity. Pharmaceuticals.

[12] Petkova-Parlapanska K., Stefanov I., Ananiev J., Georgiev T., Hadzhibozheva P., Petrova-Tacheva V., Kaloyanov N., Georgieva E., Nikolova G., Karamalakova Y. (2025). *Sambucus nigra*-Lyophilized Fruit Extract Attenuated Acute Redox-Homeostatic Imbalance via Mutagenic and Oxidative Stress Modulation in Mice Model on Gentamicin-Induced Nephrotoxicity. Pharmaceuticals.

[13] Jado J.C., Humanes B., González-Nicolás M.Á., Camaño S., Lara J.M., López B., Cercenado E., García-Bordas J., Tejedor A., Lázaro A. (2020). Nephroprotective Effect of Cilastatin against Gentamicin-Induced Renal Injury In Vitro and In Vivo without Altering Its Bactericidal Efficiency. Antioxidants.

[14] Abarova S., Alexova R., Dragomanova S., Solak A., Fagone P., Mangano K., Petralia M.C., Nicoletti F., Kalfin R., Tancheva L. (2024). Emerging Therapeutic Potential of Polyphenols from *Geranium sanguineum* L. in Viral Infections, Including SARS-CoV-2. Biomolecules.

[15] Radulović N.S., Stojković M.B., Mitić S.S., Randjelović P.J., Ilić I.R., Stojanović N.M., Stojanović-Radić Z.Z. (2012). Exploitation of the antioxidant potential of *Geranium macrorrhizum* (Geraniaceae): Hepatoprotective and antimicrobial activities. Nat. Prod. Commun..

[16] Ilić M.D., Marčetić M.D., Zlatković B.K., Lakušić B.S., Kovačević N.N., Drobac M.M. (2020). Chemical Composition of Volatiles of Eight *Geranium* L. Species from Vlasina Plateau (South Eastern Serbia). Chem. Biodivers..

[17] Venskutonis P.R., Dedonyte V., Lazutka J., Slapsyte G., Maroziene A., Nemeikaite-Ceniene A., Cenas N., Miliauskas G. (2010). A preliminary assessment of singlet oxygen scavenging, cytotoxic and genotoxic properties of *Geranium macrorrhizum* extracts. Acta Biochim. Pol..

[18] Tzanova M.T., Grozeva N.H., Gerdzhikova M.A., Todorova M.H. (2024). Composition and antioxidant potential of essential oil of *Geranium macrorrhizum* L. from different regions of Bulgaria. Bulg. Chem. Commun..

[19] Wu L., Wang L., Tian X., Zhang J., Feng H. (2020). Germacrone exerts anti-cancer effects on gastric cancer through induction of cell cycle arrest and promotion of apoptosis. BMC Complement. Med. Ther..

[20] Khajevand-Khazaei M.R., Mohseni-Moghaddam P., Hosseini M., Gholami L., Baluchnejadmojarad T., Roghani M. (2018). Rutin, a quercetin glycoside, alleviates acute endotoxemic kidney injury in C57BL/6 mice via suppression of inflammation and up-regulation of antioxidants and SIRT1. Eur. J. Pharmacol..

[21] Soodvilai S., Meetam P., Siangjong L., Chokchaisiri R., Suksamrarn A., Soodvilai S. (2020). Germacrone Reduces Cisplatin-Induced Toxicity of Renal Proximal Tubular Cells via Inhibition of Organic Cation Transporter. Biol. Pharm. Bull..

[22] Chaudhary S.C., Siddiqui M.S., Athar M., Alam M.S. (2013). Geraniol inhibits murine skin tumorigenesis by modulating COX-2 expression, Ras-ERK1/2 signaling pathway and apoptosis. J. Appl. Toxicol..

[23] Zeima N.M., EL-Gawish A.M. (2021). The Prophylactic Effect of some herbs extract on Gentamicin Induced Nephrotoxicity in Albino Rats. Egypt. J. Nutr. Health.

[24] Juan C.A., Pérez de la Lastra J.M., Plou F.J., Pérez-Lebeña E. (2021). The Chemistry of Reactive Oxygen Species (ROS) Revisited: Outlining Their Role in Biological Macromolecules (DNA, Lipids and Proteins) and Induced Pathologies. Int. J. Mol. Sci..

[25] Abu Almaaty A.H., Elmasry R.A., Farrag M.S., Althobaiti F., Aldhahrani A., Fayad E., Hussain M.A. (2021). Impact of Human Umbilical Cord Blood Mononuclear Cells on Gentamicin-Induced Renal Injury and Genotoxicity in Rats. Front. Med..

[26] Solanki S., Modi C.M., Patel H.B., Patel U.D., Singh V. (2022). Protective Effect of Polyherbal Extract on Gentamicin-Induced Renal Injury in Wistar Rats. Indian. J. Pharm. Sci..

[27] Guerreiro Í., Ferreira-Pêgo C., Carregosa D., Santos C.N., Menezes R., Fernandes A.S., Costa J.G. (2022). Polyphenols and Their Metabolites in Renal Diseases: An Overview. Foods.

[28] Albalawi R.S., Binmahfouz L.S., Hareeri R.H., Shaik R.A., Bagher A.M. (2023). Parthenolide Phytosomes Attenuated Gentamicin-Induced Nephrotoxicity in Rats via Activation of Sirt-1, Nrf2, OH-1, and NQO1 Axis. Molecules.

[29] M’hamdi Z., Davì F., Elhourri M., Amechrouq A., Mondello F., Cacciola F., Laganà Vinci R., Mondello L., Miceli N., Taviano M.F. (2024). Phytochemical Investigations, Antioxidant and Insecticidal Properties of Essential Oil and Extracts from the Aerial Parts of *Pelargonium graveolens* from Morocco. Molecules.

[30] Zor A. (2020). Genotoxicity Testing of *Geranium macrorrhizum* Extracts. Master’s Thesis.

[31] Baker B.P., Grant J.A. (2018). Geranium Oil Profile. https://hdl.handle.net/1813/56128.

[32] Chaudhary P., Janmeda P., Docea A.O., Yeskaliyeva B., Abdull Razis A.F., Modu B., Calina D., Sharifi-Rad J. (2023). Oxidative stress, free radicals and antioxidants: Potential crosstalk in the pathophysiology of human diseases. Front. Chem..

[33] Stojanović N.M., Ranđelović P.J., Simonović M., Radić M., Todorović S., Corrigan M., Harkin A., Boylan F. (2024). Essential Oil Constituents as Anti-Inflammatory and Neuroprotective Agents: An Insight through Microglia Modulation. Int. J. Mol. Sci..

[34] Ben Slima A., Ali M.B., Barkallah M., Traore A.I., Boudawara T., Allouche N., Gdoura R. (2013). Antioxidant properties of *Pelargonium graveolens* L’Her essential oil on the reproductive damage induced by deltamethrin in mice as compared to alpha-tocopherol. Lipids Health Dis..

[35] Džamić A.M., Soković M.D., Ristić M.S., Grujić S.M., Mileski K.S., Marin P.D. (2014). Chemical composition, antifungal and antioxidant activity of *Pelargonium graveolens* essential oil. J. Appl. Pharm. Sci..

[36] Lu Y., Foo L.Y. (2001). Antioxidant activities of polyphenol from sage (*Salvia officinalis*). Food Chem..

[37] Nivitabishekam S.N., Asad M., Prasad V.S. (2009). Pharmacodynamic interaction of *Momordica charantia* with rosiglitazone in rats. Chem. Biol. Interact..

[38] Brito R.G., Guimarães A.G., Quintans J.S., Santos M.R., De Sousa D.P., Badaue-Passos D., de Lucca W., Brito F.A., Barreto E.O., Oliveira A.P. (2012). Citronellol, a monoterpene alcohol, reduces nociceptive and inflammatory activities in rodents. J. Nat. Med..

[39] Lohani A., Mishra A.K., Verma A. (2019). Cosmeceutical potential of geranium and calendula essential oil: Determination of antioxidant activity and in vitro sun protection factor. J. Cosmet. Dermatol..

[40] Chalchat J.C., Petrovic S.D., Maksimovic Z.A., Gorunovic M.S. (2002). A Comparative Study on Essential Oils of *Geranium macrorrhizum* L. and *Geranium phaeum* L., *Geraniaceae* from Serbia. J. Essent. Oil Res..

[41] Ameline A., Dorland J., Werrie P.Y., Couty A., Fauconnier M.L., Lateur M., Doury G. (2023). *Geranium macrorrhizum*, a potential novel companion plant affecting preference and performance of *Myzus persicae* on sweet pepper. J. Pest Sci..

[42] Koutsaviti A., Antonopoulou V., Vlassi A., Antonatos S., Michaelakis A., Papachristos D.P., Tzakou O. (2018). Chemical composition and fumigant activity of essential oils from six plant families against *Sitophilus oryzae* (Col: Curculionidae). J. Pest Sci..

[43] Kashyap D., Mittal S., Sak K., Singhal P., Tuli H.S. (2016). Molecular mechanisms of action of quercetin in cancer: Recent advances. Tumor Biol..

[44] Dai L., Watanabe M., Qureshi A.R., Mukai H., Machowska A., Heimbürger O., Barany P., Lindholm B., Stenvinkel P. (2019). Serum 8-hydroxydeoxyguanosine, a marker of oxidative DNA damage, is associated with mortality independent of inflammation in chronic kidney disease. Eur. J. Intern. Med..

[45] Wang Y., He X., Xue M., Sun W., He Q., Jin J. (2023). Germacrone protects renal tubular cells against ferroptotic death and ROS release by re-activating mitophagy in diabetic nephropathy. Free Radic. Res..

[46] Ragab E. (2007). Geranium Plant Extracts Modulates Gamma Radiation-Induced Biochemical Hazardous Changes in Rats. Isot. Radiat. Res..

[47] Kennedy L., Sandhu J.K., Harper M.E., Cuperlovic-Culf M. (2020). Role of Glutathione in Cancer: From Mechanisms to Therapies. Biomolecules.

[48] Boadi W.Y., Amartey P.K., Lo A. (2016). Effect of quercetin, genistein and kaempferol on glutathione and glutathione-redox cycle enzymes in 3T3-L1 preadipocytes. Drug Chem. Toxicol..

[49] Zhuang S., Liu B., Guo S., Xue Y., Wu L., Liu S., Zhang C., Ni X. (2021). Germacrone alleviates neurological deficits following traumatic brain injury by modulating neuroinflammation and oxidative stress. BMC Complement. Med. Ther..

[50] Jin J., Wang Y., Zheng D., Liang M., He Q. (2022). A Novel Identified Circular RNA, mmu_mmu_circRNA_0000309, Involves in Germacrone-Mediated Improvement of Diabetic Nephropathy Through Regulating Ferroptosis by Targeting miR-188-3p/GPX4 Signaling Axis. Antioxid. Redox Signal..

[51] Zhao L.P., Wang H.J., Hu D., Hu J.H., Guan Z.R., Yu L.H., Jiang Y.P., Tang X.Q., Zhou Z.H., Xie T. (2024). β-Elemene induced ferroptosis via TFEB-mediated GPX4 degradation in EGFR wide-type non-small cell lung cancer. J. Adv. Res..

[52] Xie J., Wang H., Xie W., Liu Y., Chen Y. (2024). Gallic acid promotes ferroptosis in hepatocellular carcinoma via inactivating Wnt/β-catenin signaling pathway. Naunyn Schmiedebergs Arch. Pharmacol..

[53] Li L., Wang K., Jia R., Xie J., Ma L., Hao Z., Zhang W., Mo J., Ren F. (2022). Ferroportin-dependent ferroptosis induced by ellagic acid retards liver fibrosis by impairing the SNARE complexes formation. Redox Biol..

[54] Benoit S.W., Ciccia E.A., Devarajan P. (2020). Cystatin C as a biomarker of chronic kidney disease: Latest developments. Expert Rev. Mol. Diagn..

[55] Song J., Yu J., Prayogo G.W., Cao W., Wu Y., Jia Z., Zhang A. (2019). Understanding kidney injury molecule 1: A novel immune factor in kidney pathophysiology. Am. J. Transl. Res..

[56] Brilland B., Boud’hors C., Wacrenier S., Blanchard S., Cayon J., Blanchet O., Piccoli G.B., Henry N., Djema A., Coindre J.P. (2023). Kidney injury molecule 1 (KIM-1): A potential biomarker of acute kidney injury and tubulointerstitial injury in patients with ANCA-glomerulonephritis. Clin. Kidney J..

[57] Hewitson T.D., Smith E.R., Samuel C.S. (2014). Qualitative and quantitative analysis of fibrosis in the kidney. Nephrology.

[58] Georgieva E., Atanasov V., Kostandieva R., Tsoneva V., Mitev M., Arabadzhiev G., Yovchev Y., Karamalakova Y., Nikolova G. (2023). Direct Application of 3-Maleimido-PROXYL for Proving Hypoalbuminemia in Cases of SARS-CoV-2 Infection: The Potential Diagnostic Method of Determining Albumin Instability and Oxidized Protein Level in Severe COVID-19. Int. J. Mol. Sci..

[59] Ibrahim Y.F., Hammady S.H., Rifaai R.A., Waz S., Ibrahim M.A., Hafez H.M. (2022). Dose-dependent ameliorating effect of lipoxin A4 on gentamicin-induced nephrotoxicity in rats: The role of TNFα, TGF-β, ICAM-1, and JNK signaling. Chem. Biol. Interact..

[60] Abu Shelbayeh O., Arroum T., Morris S., Busch K.B. (2023). PGC-1α Is a Master Regulator of Mitochondrial Lifecycle and ROS Stress Response. Antioxidants.

[61] Fontecha-Barriuso M., Martín-Sánchez D., Martinez-Moreno J.M., Carrasco S., Ruiz-Andrés O., Monsalve M., Sanchez-Ramos C., Gómez M.J., Ruiz-Ortega M., Sánchez-Niño M.D. (2019). PGC-1α deficiency causes spontaneous kidney inflammation and increases the severity of nephrotoxic AKI. J. Pathol..

[62] Li J., Xu F., Li S., Xie M., Li N. (2022). Gentamicin promoted the production of CD4^+^CD25^+^ Tregs via the STAT5 signaling pathway in mice sepsis. BMC Immunol..

[63] Tsuji-Takayama K., Suzuki M., Yamamoto M., Harashima A., Okochi A., Otani T., Inoue T., Sugimoto A., Toraya T., Takeuchi M. (2008). The production of IL-10 by human regulatory T cells is enhanced by IL-2 through a STAT5-responsive intronic enhancer in the IL-10 locus. J. Immunol..

[64] Chatterjee P.K., Cuzzocrea S., Brown P.A., Zacharowski K., Stewart K.N., Mota-Filipe H., Thiemermann C. (2000). Tempol, a membrane-permeable radical scavenger, reduces oxidant stress-mediated renal dysfunction and injury in the rat. Kidney Int..

[65] Collin F. (2019). Chemical Basis of Reactive Oxygen Species Reactivity and Involvement in Neurodegenerative Diseases. Int. J. Mol. Sci..

[66] Azzam S.M., Elsanhory H.M.A., Abd El-Slam A.H., Diab M.S.M., Ibrahim H.M., Yousef A.M., Sabry F.M., Khojah E.Y., Bokhari S.A., Salem G.E.M. (2024). Protective effects of *Pelargonium graveolens* (geranium) oil against cefotaxime-induced hepato-renal toxicity in rats. Front. Toxicol..

[67] Graça V.C., Ferreira I.C.F.R., Santos P.F. (2020). Bioactivity of the *Geranium* Genus: A Comprehensive Review. Curr. Pharm. Des..

[68] El-Shamy K.A., Koriem K.M.M., Fadl N.N., El-Azma M.H.A., Arbid M.S.S., Morsy F.A., El-Zayat S.R., Hosny E.N., Youness E.R. (2019). Oral supplementation with geranium oil or anise oil ameliorates depressed rat-related symptoms through oils antioxidant effects. J. Complement. Integr. Med..

[69] Petkova-Parlapanska K., Draganova V., Georgieva E., Goycheva P., Nikolova G., Karamalakova Y. (2025). Systematic Inflammation and Oxidative Stress Elevation in Diabetic Retinopathy and Diabetic Patients with Macular Edema. Int. J. Mol. Sci..

[70] Gielis J.F., Boulet G.A., Briedé J.J., Horemans T., Debergh T., Kussé M., Cos P., Van Schil P.E. (2015). Longitudinal quantification of radical bursts during pulmonary ischaemia and reperfusion. Eur. J. Cardiothorac. Surg..

[71] Perrone S., Santacroce A., Longini M., Proietti F., Bazzini F., Buonocore G. (2018). The Free Radical Diseases of Prematurity: From Cellular Mechanisms to Bedside. Oxid. Med. Cell. Longev..

[72] Bailey D.M. (2004). Ascorbate, blood-brain barrier function and acute mountain sickness: A radical hypothesis. Wilderness Environ. Med..

[73] Goycheva P., Petkova-Parlapanska K., Georgieva E., Karamalakova Y., Nikolova G. (2023). Biomarkers of Oxidative Stress in Diabetes Mellitus with Diabetic Nephropathy Complications. Int. J. Mol. Sci..

